# Supplementary material for: The Evaluation of CYP2D6, CYP2C9, CYP2C19, and CYP2B6 Phenoconversion in Post-Mortem Casework: The Challenge of Forensic Toxicogenetics
Source: Metabolites. 2023 May 16;13(5):661. doi: 10.3390/metabo13050661 (PMC10221100; doi:10.3390/metabo13050661)
Supplement: Supplementary file 1 [file metabolites-13-00661-s001.zip › metabolites-2358157-supplementary.pdf]

Supplementary material to:

The evaluation of *CYP2D6, CYP2C9, CYP2C19*, and *CYP2B6* phenoconversion in post-mortem casework: the challenge of forensic toxicogenetics

Table S1. List of genotyped variants for *CYP2D6*, *CYP2C9*, *CYP2C19* and *CYP2B6*.

Gene symbol	dbSNP reference (variant allele)	Function
CYP2D6 [59,33]	(*CYP2D6*1*)	Wild type (Normal function)
	rs16947 (*CYP2D6**2)	Normal function
	rs35742686 (*CYP2D6*3)*	No function
	rs3892097 (*CYP2D6*4*)	No function
	rs5030655 (*CYP2D6*6*)	No function
	rs5030656 (*CYP2D6*9*)	Decreased function
	rs1065852(*CYP2D6*10*)	Decreased function
	rs28371706(*CYP2D6*17)*	Decreased function
	rs28371725(*CYP2D6*41*)	Decreased function

	rs79292917(*CYP2D6*59*)	Decreased function
CYP2C9 [60]	(*CYP2C9*1*)	Wild type (Normal function)
	rs179985(*CYP2C9**2)	Decreased function
	rs1057910(*CYP2C9**3)	No function
	rs28371686 (*CYP2C9*5*)	Decreased function
	rs28371685(*CYP2C9*11*)	Decreased function
CYP2C19 [60]	(*CYP2C19*1*)	Wild type (Normal function)
	rs4244285 (*CYP2C19**2)	No function
	rs4986893 (*CYP2C19**3)	No function
CYP2B6 [53,42]	(*CYP2C2B6*1*)	Wild type (Normal function)
	rs8192709(*CYP2B6**2)	Normal function
	rs2279343(*CYP2B6*4)*	Increased function
	rs3211371(*CYP2B6*5*)	Normal function
	rs12721655(*CYP2B6*8*)	No function

	rs3745274(*CYP2B6*9*)	Decreased function
	rs28399499(*CYP2B6*18*)	No function
	rs34826503(*CYP2B6*19*)	Decreased function
	rs34223104 (*CYP2B6*22*)	Increased function

Table S2. General parameters useful for CYP2D6, CYP2C9, CYP2C19 and CYP2B6 to determine the g-phenotype starting from the activity* score (*only CYP2D6, CYP2C9) and the p-phenotype considering the drugs found in the samples. Starting AS: activity score based on genotype. Adjusted AS: activity score adjusted according to the models described in the literature. g-phenotype: genotype-based phenotype. p-phenotype: phenoconversion-induced phenotype. IM: intermediate metabolizers, NM: normal metabolizers, PM: poor metabolizers, UM: ultrarapid metabolizers. For homogeneous classification UM was converted in EM: extensive metabolizers (for CYP2B6).

CYP2D6				
**Starting AS**	**g-phenotype**	**Inhibitor (multiplicative factor)**	**Adjusted AS**	**p-phenotype**
0	gPM	strong (0)	0	PM
		Moderate\weak (0,5)	0	PM
0 < x < 1.25	gIM	strong (0)	0	PM
		Moderate\weak (0,5)	0,13 < x < 0,5	IM
1.25 ≤ x ≤2.25	gNM	strong (0)	0	PM
		Moderate\weak (0,5)	0,63 < x < 1,13	IM
> 2.25	gUM	strong (0)	0	PM
		Moderate\weak (0,5)	>1,25	NM

CYP2C9 **and** *CYP2C19*			
**Starting AS***	**g-phenotype**	**p-phenotype with Inhibitor**	**p-phenotype with Inducer**
0 < x < 0,5	gPM	PM	PM
1 < x < 1,5	gIM	PM	NM
2	gNM	PM	UM
> 2	gUM	IM	UM
CYP2B6			
	**g-phenotype**	**p-phenotype with inhibitor**	**p-phenotype with inducer**
-	gPM	PM	IM\EM
-	gIM	PM	high IM\EM
-	gNM	low IM\PM	EM
-	gEM	high IM	EM
*only *CYP2C9*			

Table S3. **Specifications of the samples included in the study**. Case number (n) (PHG: phenoconversion), gender, age, ethnicity, Cause (CoD) and Manner of death (MoD) as well as toxicological results including ethanol of the post-mortem cases analysed.

Case n.	Gender	Age	Ethnicity	Past history	CoD	MoD	Ethanol (g/l)	Toxicological results (ng/ml)
**PHG1**	M	43	European	Unknown	Asphyxia	Suicide	-	Tramadol 904.7
**PHG2**	F	52	European	Psychiatric disease, drug abuse	Acute intoxication by multiple substances	Suicide	-	Morphine 491.6, THC 3.8, citalopram 389.3 nordazepam 189.9 diazepam 222.6 quetiapine 451.5
**PHG3**	F	37	European	Drug abuse, epilepsy	Acute intoxication by morphine	Accident	-	Morphine 500, dextromethorphan, hydromorphone, clotiapine 26

**PHG4**	F	55	European	Psychiatric disease	Acute intoxication by multiple substances	Suicide	-	Codeine 749, tramadol 788, oxycodon 352, O-desmethyltramadol, oxymorphone, paracetamol
**PHG5**	F	32	European	-	Acute intoxication by multiple substances	Accident	-	Cocaine 6,393, methadone 1,679, chlorpromazine 60.2, desalkylfluorazepam 64.8, diazepam 40 ng/ml, nordazepam 53.5 mirtazapine 186.6
**PHG6**	M	37	European	Unknown	Acute intoxication by multiple substances	Accident	-	Morphine 315, codeine 46, paroxetine 35.5, norketamine, dextromethorphan, hydromorphone
**PHG7**	M	44	European	Unknown	Acute intoxication by multiple substances	Accident	0.18	Cocaine 3,796, mirtazapine > 2000, paroxetine 1,217.9, lorazepam 277.6 trazodone 2,158.6
**PHG8**	M	31	European	Psychiatric disease	Blunt trauma	Accident	-	Clozapine 943.3, haloperidol 21.1, diazepam 198.6, nordazepam 287.7
**PHG9**	M	51	European	Hepatitis C	Blunt trauma	Accident	2.34	Amitriptyline, nortriptyline
**PHG10**	M	36	European	Unknown	Burning	Suicide	-	Diazepam 153.6, nordazepam 257.7 fluoxetine < 600, desalchylflurazepam 206.5, trazodon 176.8, norfluoxetine
**PHG11**	M	35	European	Psychiatric disease, hepatitis C, neurological disease	Acute intoxication by multiple substances	Accident	-	Cocaine 2,265, methadone 255.6, diazepam 15.9, venlafaxine 569.6, o-desmethylvenlafaxine 194.3
**PHG12**	M	69	European	Psychiatric disease	Blunt trauma	Accident	0.80	Paroxetine 269, ketamine, midazolam 72
**PHG13**	M	30	Mediterranean	Unknown	Acute intoxication by multiple substances	Accident	0.67	Methadone 1,065, morphine 19, diazepam 187, nordazepam 192, temazepam traces, olanzapine >145; citalopram >259
**PHG14**	F	47	European	Psychiatric disease	Blunt trauma	Accident	-	Methadone 347, fluoxetine 3,235, nordiazepam, olanzapine 957.14, paracetamol, trazadon 107.2, diazepam, desmethylolazapine 25.9, clotiapine traces
**PHG15**	M	35	Mediterranean	-	Acute intoxication by	Accident	-	Methadone 441.9, promazine 44, diazepam traces, mirtazapine 5.9, tramadol traces

					multiple substances			
**PHG16**	M	53	Arabian Peninsula	Psychiatric disease, drug abuse	Sudden cardiac death	Natural	2.76	Nordiazepam 42.1, lidocaine, mirtazapine 130.4, metoprolol, amiodarone 29.9, diazepam 98.3, clotiapine 10.7
**PHG17**	M	40	European	Psychiatric disease	Asphyxia	Accident	-	7-aminoclonazepam 54.1, levomepromazine 36.7, quetiapine 91.3, norquetiapine 224.4, valproic acid 4.8 microgram/ml
**PHG18**	M	80	European	-	Sudden cardiac death	Natural	-	Amitriptyline 126.0, nortriptyline 64.2, sertraline 208.1, N-desmethylsertraline 192.2, promazine traces, trazodon traces, lorazepam 328.8
**PHG19**	M	85	European	-	Blunt trauma	Accident	-	Fentanyl, lidocaine, paracetamol, promazine
**PHG20**	M	57	European	-	Sudden cardiac death	Natural	0.11	Paroxetine 259.2, alprazolam 26.4
**PHG21**	M	44	European	Psychiatric disease	Acute intoxication by olanzapine		-	Olanzapine > 2000 ng/m; desmethylolanzapine 92.1 ng/ml
**PHG22**	M	58	European	Drug abuse	Acute intoxication by multiple substances	Accident	-	Methadone 297, levomepromazine, 83 paroxetine 126.1, quetiapine, norquetiapine, ephedrine, pseudoephedrine
**PHG23**	M	43	European	Psychiatric disease, epilepsy	Acute intoxication by multiple substances	Suicide	-	Methadone 77.7, morphine 1,069.2, nordazepam 144.7, diazepam 33.7, oxazepam 49.8, temazepam 23.2
**PHG24**	M	44	European	-	Acute intoxication by multiple substances	Accident	-	Cocaine 1,380, methadone 80, morphine 582, codeine 43
**PHG25**	M	41	European	-	Acute intoxication by methadone	Accident	0.67	Methadone 437.14
**PHG26**	F	32	European	Drug abuse	Acute intoxication by multiple substances	Accident	-	Cocaine 5,915, methadone 1,523, chlorpromazine 45.7, 52.6, diazepam 53 ng/ml, nordazepam 46.2 mirtazapine 157.6

**PHG27**	M	26	European	Drug abuse	Acute intoxication by methadone	Accident	-	Methadone 905.9
**PHG28**	M	25	European	-	Acute intoxication by multiple substances	Accident	-	Methadone 33, morphine 158
**PHG29**	M	56	European	Drug abuse, HIV	Acute intoxication by multiple substances	Accident	-	Cocaine 137, methadone 506, diazepam 999, nordazepam 910
**PHG30**	M	31	European	Psychiatric disease, alcohol abuse	Acute intoxication by multiple substances	Accident	-	Methadone 342.5
**PHG31**	M	30	Mediterranean	Alcohol abuse	Acute intoxication by multiple substances	Accident	0.67	Methadone 1,065, morphine 52, diazepam 187, nordazepam 192, temazepam traces, olanzapine >145, citalopram >259
**PHG32**	F	31	European	Drug abuse	Blunt trauma	Suicide	-	Methadone 1,112.4, THC 2.1, diazepam 60.5, nordazepam 78.5, mirtazapine 11.4
**PHG33**	M	28	Arabian Peninsula	Psychiatric disease, drug abuse	Acute intoxication by methadone	Accident	-	Methadone 441.9, promazine 44, diazepam traces, mirtazapine 5.9, tramadol traces
**PHG34**	M	40	European	Drug abuse	Acute intoxication by multiple substances	Accident	-	Cocaine 138, methadone 503, paroxetine 43, trazodone 291, aripiprazole 52
**PHG35**	M	46	European	Drug abuse	Acute intoxication by multiple substances	Accident	-	Methadone 272, morphine 621.8, codeine 46.3, phenacetin

Table S4. Genotype, g-phenotype and p-phenotype for CYP2D6, CYP2C9, CYP2C19, CYP2B6. Case number (n), PHG: phenoconversion , g-phenotype: phenotype based on genotype, p-phenotype: phenoconversion-induced phenotype. na: not applicable because both inhibitors and inducers are present, nd=not determined, *=samples containing inhibitors (lidocaine and olanzapine) but information on their strength is lacking in the literature.

Case n.	Genotype CYP2D6	g-phenotype CYP2D6	p-phenotype CYP2D6	GenotypeCYP2C9	g-phenotype CYP2C9	p-phenotype CYP2C9	Genotype CYP2C19	g-phenotype CYP2C19	p-phenotype CYP2C19	GenotypeCYP2B6	g-phenotype CYP2B6	p-phenotype CYP2B6
**PHG1**	*1*2	gNM	IM	*1*1	gNM	PM	nd	nd	nd	nd	nd	nd
**PHG2**	*4*5	gPM	PM	*1*2	gIM	NM	*1*1	gNM	PM	*1*6	gIM	high-IM\EM
**PHG3**	*1*1	gNM	NM	*1*1	gNM	NM	*1*2	gIM	NM	*1*5	gNM	NM
**PHG4**	*1*2	gNM	NM	*1*1	gNM	NM	*1*1	gNM	NM	*1*1	gNM	NM
**PHG5**	*1*1 x3	gUM	PM	*1*1	gNM	NM	*1*1	gNM	PM	*1*6	gIM	high-IM\EM
**PHG6**	*1*1	gNM	PM	*1*2	gIM	PM	*1*1	gNM	NM	*1*4	gEM	high-IM
**PHG7**	*1*1 x3	gUM	PM	*1*2	gIM	NM	nd	nd	nd	*1*9	gIM	PM
**PHG8**	*10*10	gIM	PM	*1*1	gNM	NM	nd	nd	nd	*1*4	gEM	EM
**PHG9**	*2*2 x3	gUM	UM	*1*1	gNM	NM	nd	nd	nd	*1*4	gEM	EM
**PHG10**	*2*2	gNM	PM	*1*2	gIM	PM	nd	nd	nd	*1*6	gIM	high-IM\EM
**PHG11**	*2*2	gNM	PM	*1*1	gNM	NM	*1*2	gIM	PM	*1*6	gIM	high-IM\EM
**PHG12**	*1*41 x3	gUM	PM	*2*3	gPM	PM	*1*2	gIM	NM	*1*6	gIM	PM
**PHG13**	*1*2 x4	gUM	IM	*1*1	gNM	PM	*1*1	gNM	PM	*1*1	gNM	EM
**PHG14**	*2*2	gNM	PM	*1*1	gNM	PM	*1*1	gNM	PM	*1*6	gIM	high-IM\EM

**PHG15**	*1*1	gNM	IM	*1*1	gNM	NM	*1*1	gNM	PM	*1*5	gNM	EM
**PHG16**	*41*41	gIM	IM	*1*1	gNM	PM	*1*1	gNM	PM	*1*4	gEM	EM
**PHG17**	*1*4	gIM	PM	*1*2	gIM	PM	*1*1	gNM	PM	*1*4	gEM	EM
**PHG18**	*4*41	gIM	IM	*1*1	gNM	PM	*1*2	gIM	PM	*1*6	gIM	PM
**PHG19**	*2*41	gNM	IM or PM*	*1*2	gIM	NM	*1*2	gIM	NM	*1*7	gIM	IM
**PHG20**	*4*10	gIM	PM	*1*3	gIM	PM	*1*1	gNM	NM	*1*1	gNM	lowIM\PM
**PHG21**	*1*1	gNM	IM or PM*	*1*1	gNM	NM	*1*1	gNM	PM	*1*1	gNM	NM
**PHG22**	*2*2	gNM	PM	*1*1	gNM	PM	*1*1	gNM	NM	*1*1	gNM	na
**PHG23**	*2*4	gIM	PM	*1*2	gIM	na	*1*1	gNM	na	*1*5	gNM	EM
**PHG24**	*1*4	gIM	IM	*1*1	gNM	UM	*1*1	gNM	PM	*1*5	gNM	EM
**PHG25**	*1*4	gIM	IM	*1*1	gNM	NM	*1*1	gNM	NM	*1*7	gIM	high-IM\EM
**PHG26**	*1*1	gNM	PM	*1*1	gNM	NM	*1*1	gNM	PM	*1*6	gIM	high-IM\EM
**PHG27**	*2*41	gNM	IM	*1*1	gNM	NM	*2*2	gPM	NM	*2*5	gNM	EM

**PHG28**	*1*4	gIM	IM	*1*1	gNM	NM	*1*1	gNM	NM	*1*7	gIM	high-IM\EM
**PHG29**	*1*1	gNM	PM	*1*1	gNM	NM	*1*1	gNM	PM	*1*1	gNM	EM
**PHG30**	*2*4	gIM	IM	*1*2	gIM	NM	nd	nd	nd	*1*22	gEM	EM
**PHG31**	*1*2	gNM	IM	*1*1	gNM	PM	nd	nd	nd	*1*1	gNM	EM
**PHG32**	*1*4	gIM	IM	*2*2	gIM	PM	*1*1	gNM	PM	*1*6	gIM	na
**PHG33**	*1*2	gNM	IM	*1*1	gNM	NM	*1*2	gIM	PM	*1*5	gNM	EM
**PHG34**	*2*2 x4	gUM	PM	*1*2	gIM	PM	*1*1	gNM	PM	*1*6	gIM	na
**PHG35**	*1*1	gNM	IM	*1*1	gNM	NM	*1*1	gNM	NM	*1*4	gEM	EM
